# High temperature accelerates growth of aerobic anoxygenic phototrophic bacteria in seawater

**DOI:** 10.1002/mbo3.710

**Published:** 2018-07-27

**Authors:** Yuki Sato‐Takabe, Koji Hamasaki, Satoru Suzuki

**Affiliations:** ^1^ Center for Marine Environmental Studies Ehime University, Matsuyama Ehime Japan; ^2^ Atmosphere and Ocean Research Institute The University of Tokyo Kashiwa Japan

**Keywords:** aerobic anoxygenic phototrophic bacteria, grazing effect, growth rate, temperature

## Abstract

Temperature is an important controlling factor in the growth activity of all microorganisms. Aerobic anoxygenic phototrophic (AAP) bacteria actively grow in the ocean and are known as one of the main driving forces in organic matter cycling in surface seawater environments. Whether temperature change affects AAP bacteria activity from an ecological viewpoint remains an open question. To date, no known studies have reported the effect of temperature change on AAP bacteria growth in the ocean. We here show that the growth rate of AAP bacteria exceeded that of other bacterial types at high water temperatures in the absence of grazers. The slope of the regression line of the net growth rate of AAP bacteria as a function of water temperature was the same as that for non‐AAP bacteria at all temperatures (10, 20, and 30°C); however, when grazers were eliminated, it was 4.7 times higher than that of non‐AAP bacteria. This result suggests that AAP bacteria are more responsive to water temperature increases than other bacteria and that AAP bacteria might become more dominant than other bacteria under elevated water temperatures.

## 1 INTRODUCTION

Temperature is an important controlling factor of the physiological activity of bacterial community. Bacterial growth and respiratory rates show temperature dependence (Pomeroy & Wiebe, 2001; Robinson, 2008). In addition to biochemical responses, community structure also changes with temperature (Crump & Hobbie, 2005; Hewson, Steele, Capone, & Fuhrman, 2006; Lee & Fuhrman, 1991; Pinhassi & Hagström, 2000; Sjöstedt, Hagström, & Zweifel, 2012). Previous studies have shown that environmental conditions have varied effects on the population abundance of specific bacterial groups (Crump & Hobbie, 2005; Pinhassi & Hagström, 2000).

Aerobic anoxygenic phototrophic (AAP) bacteria are bacteriochlorophyll *a*‐containing bacteria that utilize both phototrophy and heterotrophy for energy gain, with heterotrophy usually being the main system (Beatty, 2002) and gains by light being minimal (Ferrera, Sánchez, Kolářová, Koblížek, & Gasol, 2017; Kirchman & Hanson, 2013). The AAP bacteria are known to be widely distributed in open and coastal oceans (Cottrell & Kirchman, 2009; Kolber et al., 2001; Lami et al., 2007; Lamy et al., 2011; Schwalbach & Fuhrman, 2005; Sieracki, Gilg, Their, Poulton, & Goericke, 2006). The abundance of AAP bacteria in the microbial community is reported to be as high as 24% (Lami et al., 2007; Sato‐Takabe et al., 2016), and we reported the high contribution of AAP bacteria in organic carbon transport from bacteria to the next trophic level in coastal oceans (Sato‐Takabe et al., 2016). As AAP bacteria reportedly showed higher growth rate than other heterotrophic bacteria (Ferrera, Gasol, Sebastián, Hojerová, & Koblížek, 2011; Koblížek, Mašín, Ras, Poulton, & Prášil,^2007^; Stegman, Cottrell, & Kirchman, 2014), we hypothesized that if the activity and growth of AAP bacteria are affected by a change in water temperature, the carbon flux to protists should also be changed in the microbial loop.

In this study, we examined the growth of AAP bacteria compared to other bacterial members at various temperatures in enclosed microcosms. Microcosms with and without grazers were designed to observe the net and intrinsic growth of the bacterial groups.

## 2 EXPERIMENTAL PROCEDURE

### 2.1 Seawater used for microcosm studies

Surface seawater was collected on June 11, 2014 in a coastal aquaculture area (32°56′38.37″N, 132°30′40.37″E) located about 200 m offshore in the Uwa Sea, along the south western coast of Shikoku Island, Japan, and having a water depth of approximately 50 m. Water was collected with a Niskin sampling system and transported to the laboratory within 4 hr in acid‐washed 500‐ml polycarbonate bottles rinsed three times with seawater. Water temperature, salinity, and electrical conductivity were measured using a hand‐held multimeter (pH/Cond Meter D‐54, HORIBA, Japan; Hand‐Held Refractometer ATC‐S/Mill‐E, ATAGO, Japan).

### 2.2 Microcosm setting

Water was collected in a 10‐L bottle and allocated to 500‐ml Nalgene bottles for incubation. Microcosms were prepared with unfiltered seawater, which contained grazers, or seawater prefiltered through a 0.8‐μm Nuclepore membrane filter (Advantec, Japan) to remove grazers, and bottles were incubated in triplicate at 30, 20, and 10°C under a light regime of dark (12 hr) and light (12 hr) at 5–10 μmol photons per m^2^/s. At 24, 48, and 72 hr, the bottles were mixed and opened, and 30 ml of water was removed from each bottle for analysis. The bottles were mixed before removing the water sample. Both treatments were expected to include viruses, as the filtration to remove grazers was not effective for removing viruses.

Almost all AAP bacterial cells in both unfiltered whole and filtered with 0.8 μm filter seawater were free‐living (data not shown) and not particle‐attached.

### 2.3 Enumeration of total bacteria and AAP bacteria

From each daily sample, 10 ml subsamples were fixed with neutral formalin overnight at 4°C in the dark (1.0% final concentration), and total bacterial abundance was determined by epifluorescence microscopy. In brief, the formalin‐fixed samples were filtered through Nuclepore black polycarbonate membrane filters (0.2 μm in pore size) under gentle vacuum (≤0.03 MPa). The filters were dried and then stained with 4′,6‐diamidino‐2‐phenylindole (DAPI) prepared at 1 μg/ml in a 3:1 mixture of Citifluor AF1 (Citifluor Ltd., United Kingdom) to Vectashield (Vector Labs, Canada).

**Figure 1:**
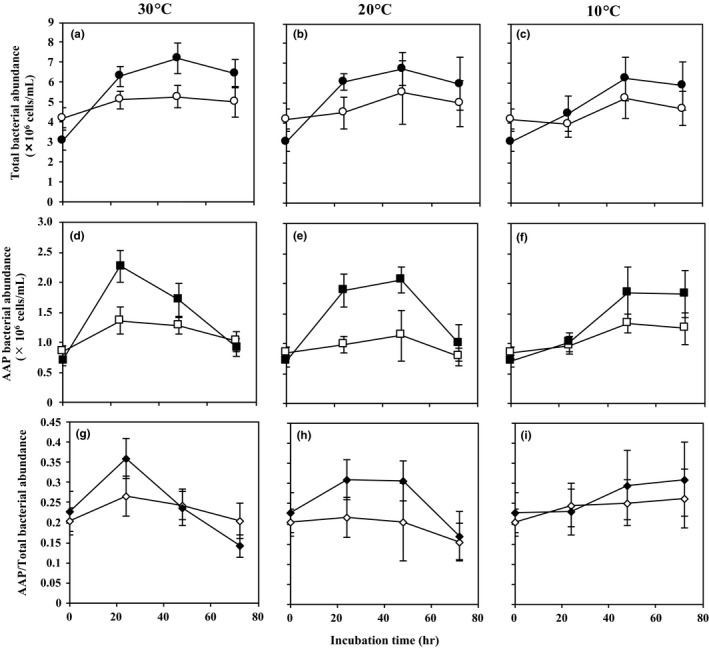
Changes over time in bacterial cell abundance of total bacteria (a–c) and aerobic anoxygenic phototrophic (AAP) bacteria (d–f). (g–i) AAP bacteria/total. Incubation was conducted at temperatures 30, 20, and 10°C. Values are shown separately for with grazers (open symbols) and without grazers (closed symbols)

**Figure 2:**
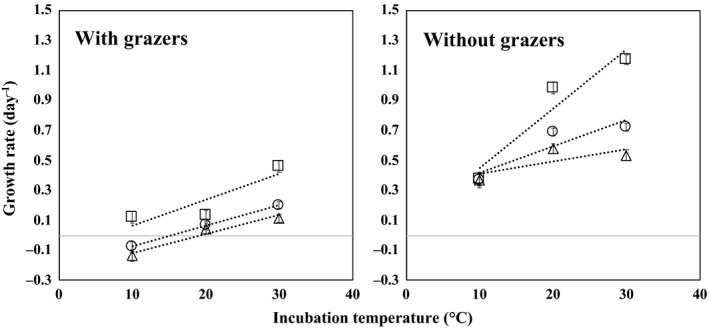
Relationship between net growth rates and incubation temperatures. Aerobic anoxygenic phototrophic (AAP) bacteria (squares), non‐AAP bacteria (triangles), and total bacteria (circles) with grazers (left) and without grazers (right)

Bacterial cells were enumerated on images taken on a Zeiss Axioplan 2 epifluorescence microscope (Carl Zeiss, Germany) equipped with a mercury lamp (USH‐102D, Ushio, Japan) and a Photometrics CH‐250 cooled, slow scan, infrared (IR)‐sensitive CCD camera (iKon‐M, ANDOR, United Kingdom) and connected to a Windows PC, as described previously (Sato‐Takabe et al., 2015, 2016). The following three epifluorescence filter sets were used: (a) BChl *a* (excitation 400–530 nm, emission >850 nm long pass, RG850, Edmund Optics, USA, >650 nm dichroic, XF‐2072, Omega Optical); (b) Chl *a* (excitation 546 ± 12 nm, emission >590 nm long pass, >580 nm dichroic, Zeiss Filter set 15, 488015–0000, Carl Zeiss); and (c) DAPI (excitation 365 ± 12 nm, emission >397 nm long pass, >395 nm dichroic, Zeiss Filter set 01, 488001–0000, Carl Zeiss). First, the total DAPI‐stained bacteria were recorded with the DAPI filter set (100 ms exposure). Then, Chl *a* autofluorescence was recorded to identify Chl *a*‐containing organisms with the Chl *a* filter set (100 ms exposure). In conclusion, IR emission (>850 nm) images were captured with the BChl *a* filter set to show both AAP bacteria and phytoplankton (10 s exposure). In general, between 10 and 15 sets of images (3–5 sets for each bottle) were acquired from each DAPI‐stained filter. Error bars in Figures 1 and 2 were calculated based on analysis of 10 and 15 sets of images, respectively. The acquired images were saved and semimanually analyzed with the aid of MetaMorph software (Molecular Device) to distinguish heterotrophic bacteria, *Synechococcus*, and AAP bacteria. Following image acquisition, the contrast and brightness of images were manipulated using the imaging software Meta Morph (Molecular Device) with the “Top Hat” process to extract cell images from the background. AAP bacterial cells were identified as having DAPI and IR fluorescence but not Chl *a* fluorescence. Cell abundance for total bacteria (total count of 511–1872 cells per sample) and AAP bacteria (total count of 124–598 cells per sample) was taken as the average values for all cells measured.

## 3 RESULTS AND DISCUSSION

The collected seawater had a temperature, pH, salinity, and conductivity of 24.3°C, 8.26, 30.3, and 4.59 S/m, respectively. Microcosms were prepared with (a) nonfiltered seawater containing bacteria and grazers or (b) 0.8 μm‐filtered seawater to remove grazers of bacteria, and all microcosms were incubated at one of three different temperatures. The numbers of total bacteria and AAP bacteria were reduced about 73%–82% in the filtered treatment. In the present microcosm setup, abiotic parameters including salinity and dissolved oxygen (DO) were not monitored during the course of the experiment. However, these abiotic parameters would be expected to remain stable during the experiment duration. Sato‐Takabe et al. (2016) reported that the DO reaches a saturated level of around 150–200 mmol O_2_ per m^3^ in summer near aquaculture areas of the Uwa Sea where seawater was collected for the present microcosm and a calculated bacterial respiration rate of 5 mmol O_2_ per m^3^/day in coastal mesocosms (Williams, 1981). This suggests that oxygen available at the start of the experimental period was not depleted and the probability of development of anaerobic anoxygenic species is negligible. An enclosed small batch culture, such as in the present microcosm, could be considered to be susceptible to the “bottle effect,” but Hammes, Vital, and Egli (2010) reported that this effect is not observed in short‐term (<5‐days) batch incubations.

Time course changes in the abundances of total bacteria and AAP bacteria, and the cell ratio of AAP bacteria to total bacteria is shown in Figure 1. In the presence of grazers, cell abundance of total bacteria was similar (4.2 × 10^6^–5.1 × 10^6^ cells/ml) at all temperatures (10–30°C) at 24 hr (Figure 1a–c); however, in the absence of grazers, the cell number increased from 3.1 × 10^6^ to 6.3 × 10^6^ cells/ml in 24 hr at 30°C (Figure 1a). A similar trend was observed at 20°C (Figure 1b), and growth at 10°C was slower than at 20 and 30°C (Figure 1c). In the presence of grazers, AAP bacterial abundance increased slightly from 8.5 × 10^5^ to 1.4 × 10^6^ cells/ml in 24 hr at 30°C (Figure 1d), and abundances at 20 and 10°C were similar (Figure 1e,f). Without grazers, AAP bacteria increased from 7.0 × 10^5^ to 2.3 × 10^6^ cells/ml at 30°C at 24 hr (Figure 1d), but cell numbers at 20 and 10°C were lower (Figure 1e,f). These results suggest that top‐down control by grazers suppresses growth of both total and AAP bacteria. Cell abundances in the absence of grazers increased more markedly from 0 to 24 hr at 30 and 20°C for both total and AAP bacteria than at low temperature (10°C), indicating that the abundance of the bulk bacterial community, including AAP bacteria, increases at higher temperatures. The cell abundance ratio of AAP bacteria to total bacteria with grazers increased from 20% to 27% in 24 hr at 30°C, but in the absence of grazers, the ratio increased to 36% (Figure 1g). A similar trend was observed at 20°C (Figure 1h), but no change in the ratio was observed at 10°C (Figure 1i). The pattern of changes in AAP bacteria suggested that AAP bacteria could grow more rapidly than other bacteria (non‐AAP bacteria) at high temperature.

Bacterial growth rate was calculated on the assumption that it follows a model of exponential growth. Growth rates of AAP bacteria were statistically significantly higher than those of non‐AAP bacteria for all temperature and grazer status conditions at 30 and 20°C (Figure 2) based on two‐tailed *t* test. Both net and intrinsic growth rates (with and without grazers, respectively) of AAP bacteria were significantly higher than those of non‐AAP bacteria at 30 and 20°C (*p* < 0.001, two‐tailed *t* test, Figure 2). Only in the case of without grazers at 10°C was the growth rate not significantly different. This result (higher growth rate for AAP bacteria than non‐AAP bacteria) in the present study was similar to results of previous studies (Ferrera et al., 2011; Koblížek et al., 2007), in which the growth rate of AAP bacteria was 0.72–2.13 per day in the Atlantic Ocean (Koblížek et al., 2007) and 0.3–3.7 per day in manipulation experiments using water collected from the Mediterranean Sea (Ferrera et al., 2011). The maximum growth rate of AAP bacteria in the present study (0.12–1.17 per day) was lower than in previous studies. These differences can be attributed to spatio‐temporal variation of samples used in each of the experiments and differences in experimental conditions.

The relationship between growth rate and temperature is also shown in Figure 2, and parameters of linear regression (slope and standard error) for total bacteria, AAP bacteria, and non‐AAP bacteria are shown in Table 1. A positive slope in all cases indicates the temperature dependence of the growth rate. The slope of the regression line was greater for AAP bacteria than for non‐AAP bacteria in the absence of grazers. The difference in growth rate between AAP and non‐AAP bacteria in the absence of grazers increased with increasing temperature, suggesting that AAP bacteria were more responsive to elevated temperature than were non‐AAP bacteria. Further, much lower growth rates for both AAP and non‐AAP bacteria with grazers than for without grazers suggest top‐down control of abundance. AAP bacteria were bigger in size (Lami et al., 2007; Sato‐Takabe et al., 2015, 2016; Sieracki et al., 2006) and bigger bacteria are preferentially grazed by heterotrophic protists in the ocean (Anderson, Larsson, & Hagstrom, 1986; Gonzalez, Sherr, & Sherr, 1990). We could reasonably hypothesize that the dominant growth of AAP bacteria at high temperatures (in the present study) and preferential grazing on AAP bacteria leads to AAP bacteria making a significant contribution of AAP bacteria to carbon cycling through microbial food webs.

**Table 1:** Slope (standard error of slope) between growth rate and temperature for total bacteria, aerobic anoxygenic phototrophic (AAP) bacteria, and non‐AAP bacteria, in this study

Treatment group	With grazers	Without grazers
Total bacteria	0.0138 (0.0002)	0.0175 (0.0082)
AAP bacteria	0.0171 (0.0090)	0.0397 (0.0120)
Non‐AAP bacteria	0.0126 (0.0031)	0.0084 (0.0074)

Bacterial abundance and growth rate were positively correlated with increasing temperature in an earlier study (Shiah & Ducklow, 1994). Other reports also showed the effect of temperature on bacterial abundance, growth, and community structure (Crump & Hobbie, 2005; Hewson et al., 2006; Lee & Fuhrman, 1991; Pinhassi & Hagström, 2000; Pomeroy & Wiebe, 2001; Robinson, 2008; Sjöstedt et al., 2012). Sato‐Takabe et al. (2016) reported seasonal dynamics of total and AAP bacterial abundances in aquaculture areas similar to the area sampled in the present study and found that higher AAP bacterial abundance tended to coincide with higher water temperature, whereas the abundance of total bacteria was not correlated with water temperature. Generally speaking, temperature is not always a primary factor in determining bacterial growth, but rather some other factors such as salinity, oxygen, organic, and inorganic nutrients can be alternative controlling factors. In particular, nutrients dynamics would be crucial to grasp the experimental conditions such as in the present microcosm. Previous studies reported that AAP bacteria grow rapidly in the ocean and estuarine regions (Ferrera et al., 2011; Koblížek et al., 2007; Stegman et al., 2014), but there was no mention of the effect of temperature. The results of the present study suggest a prominent response of AAP bacteria to temperature change, which could explain the seasonal temperature dependence of abundance observed in our previous study and support the hypothesis that temperature is an important controlling factor of AAP bacterial dynamics in natural seawater environments. It was implied that AAP bacteria might become more active than other bacteria in elevated water temperatures in the future.

Further studies are needed in order to accurately evaluate the effect of temperature change on AAP bacteria. Future experiments should include quantitative PCR to confirm trends observed by microscopy and measurement of flux of gas exchange. In addition, most importantly, as only a single microcosm was used in the present study, the microcosm experiment should be repeated in different seasons and different sites in order to gather data sufficient to draw conclusions about the effect of temperature change on bacterial communities. The present study is the first report to demonstrate that AAP bacteria are more responsive to water temperature increases than other bacteria. However, the experiments with a new experimental setups and replication should be conducted to reach more robust and accurate conclusions.

## AUTHOR CONTRIBUTION

YS‐T: planning, experiment, and writing manuscript. KH: microscope and writing manuscript. SS: writing manuscript.

## CONFLICT OF INTEREST

The contributing authors declare no conflict of interest.

## DATA ACCESSIBILITY

The contributing authors adhere to all policies on sharing data and materials described in the guidelines for authors.
